# Prognostic significance of catalase expression and its regulatory effects on hepatitis B virus X protein (HBx) in HBV-related advanced hepatocellular carcinomas

**DOI:** 10.18632/oncotarget.2625

**Published:** 2014-10-24

**Authors:** Mi-Young Cho, Jae Youn Cheong, Wonchung Lim, Sujin Jo, Youngsoo Lee, Hee-Jung Wang, Kyou-Hoon Han, Hyeseong Cho

**Affiliations:** ^1^ Department of Biochemistry and Molecular Biology, Ajou University School of Medicine, The Graduate School, Ajou University, Suwon, Korea; ^2^ Department of Biomedical Sciences, The Graduate School, Ajou University, Suwon, Korea; ^3^ Department of Gastroenterology, Ajou University Hospital, Ajou University School of Medicine, Suwon, Korea; ^4^ Genomic Instability Research Center, Ajou University School of Medicine, Suwon, Korea; ^5^ Department of Surgery, Ajou University Hospital, Ajou University School of Medicine, Suwon, Korea; ^6^ Biomedical Translational Research Center, Korea Research Institute of Bioscience and Biotechnology, Daejeon, Korea; ^7^ Current address: Department of Sports Medicine, Cheongju University, Cheongju, Korea

**Keywords:** HBx, Liver cancer, Catalase, Cysteine, ROS

## Abstract

Hepatitis B virus X protein (HBx) plays a role in liver cancer development. We previously showed that ROS increased HBx levels and here, we investigated the role of antioxidants in the regulation of HBx expression and their clinical relevance. We found that overexpression of catalase induced a significant loss in HBx levels. The cysteine null mutant of HBx (Cys^−^) showed a dramatic reduction in its protein stability. In clonogenic proliferation assays, Huh7-X cells produced a significant number of colonies whereas Huh7-Cys^−^ cells failed to generate them. The Cys at position 69 of HBx was crucial to maintain its protein stability and transactivation function in response to ROS. Among 50 HBV-related hepatocellular carcinoma (HCC) specimens, 72% of HCCs showed lower catalase levels than those of surrounding non-tumor tissues. In advanced stage IV, catalase levels in non-tumor tissues were increased whereas those in tumors were further reduced. Accordingly, patients with a high T/N ratio for catalase showed significantly longer survival than those with a low T/N ratio. Together, catalase expression in HCC patients can be clinically useful for prediction of patient survival, and restoration of catalase expression in HCCs could be an important strategy for intervention in HBV-induced liver diseases.

## INTRODUCTION

Hepatocellular carcinoma (HCC) is a common malignancy, accounting for nearly one million deaths worldwide every year [1]. Chronic infection with hepatitis B virus (HBV) is a major predisposing factor for the development of HCC. Vaccination of newborns against HBV is an effective preventive measure; however, approximately 54% of HCC cases worldwide are still associated with chronic HBV infection [2]. Surgical resection, percutaneous ablation and liver transplantation are major curative modalities, but they are limited to patients with early HCC. A large proportion of patients is diagnosed with advanced stages of HCC and therefore has fewer treatment options. Understanding the molecular mechanism by which HBV infection increases the incidence of HCC could lead to strategies for prevention and therapeutic intervention in the diseases.

HBV, a member of the *Hepadnaviridae* family, contains a 3.2-kb genome that encodes four overlapping open reading frames (ORFs). The molecular etiology of HBV-induced HCC remains unclear; however, the multifunctional HBV X protein (HBx) has implicated in cancer development. HBx plays an important role in the maintenance of viral replication, and directly stimulates several cellular kinases involved in cell proliferation and transformation [3, 4]. HBx residing in mitochondria triggers chronic inflammatory responses through activation of the nuclear factor kappa B (NF-κB) signaling pathway as well as induction of interleukins and cyclooxygenase-2 (COX-2) expression [5, 6]. Moreover, HBx in hepatocytes is largely responsible for lipid peroxidation and hepatic steatosis [7, 8], aggravating oxidative liver injury. Thus, reducing or eliminating HBx protein in HBV-infected patients represents an attractive strategy for intervening in disease progression. To date, several cellular regulators such as DDB1 (Damaged DNA-Binding protein 1), PIN1 (Peptidyl-prolyl cis/trans isomerase NIMA interacting 1) and SIAH1 (Seven in absentia homolog 1) [9-11] have been proposed to control HBx protein levels. However, their clinical relevance to disease progression remains largely unknown.

Oxidative stress has emerged as a central player in the development of pathological diseases. In HBV-infected livers, oxidative stress activates hepatic stellate cells, and chronic activation of stellate cells not only triggers fibrogenesis but also stimulates proliferation of hepatocytes, increasing the likelihood of HCC development. We previously showed that an increase of reactive oxygen species (ROS) levels in cells dramatically enhanced the stability of HBx protein [12]. Moreover, HBx-positive hepatocytes were mainly found in the periportal region where necroinflammatory activity is high [13]. Since HBx also increases intracellular ROS level through mitochondrial damage [14], it is possible that a feedback loop develops between HBx and ROS under chronic oxidative stress conditions. Thus, intervening in this feedback loop would be an effective way to prevent further disease progression. Antioxidant molecules such as catalase, superoxide dismutase (SOD) and glutathione peroxidase decrease oxidative stress; in fact, compensatory up-regulation of MnSOD has been found under oxidative stress conditions [15].

In the present study, we investigated the role of antioxidants in regulating HBx expression and assessed their clinical relevance in HBV-related HCC tissues. We found that catalase dramatically decreased HBx levels and cysteine residues of HBx are important for stability of HBx protein. In addition, we found that relative catalase expression in tumors compared to normal liver tissues can be clinically useful for predicting patient survival.

## RESULTS

### HBx protein levels are decreased by catalase and MnSOD

**Figure 1:**
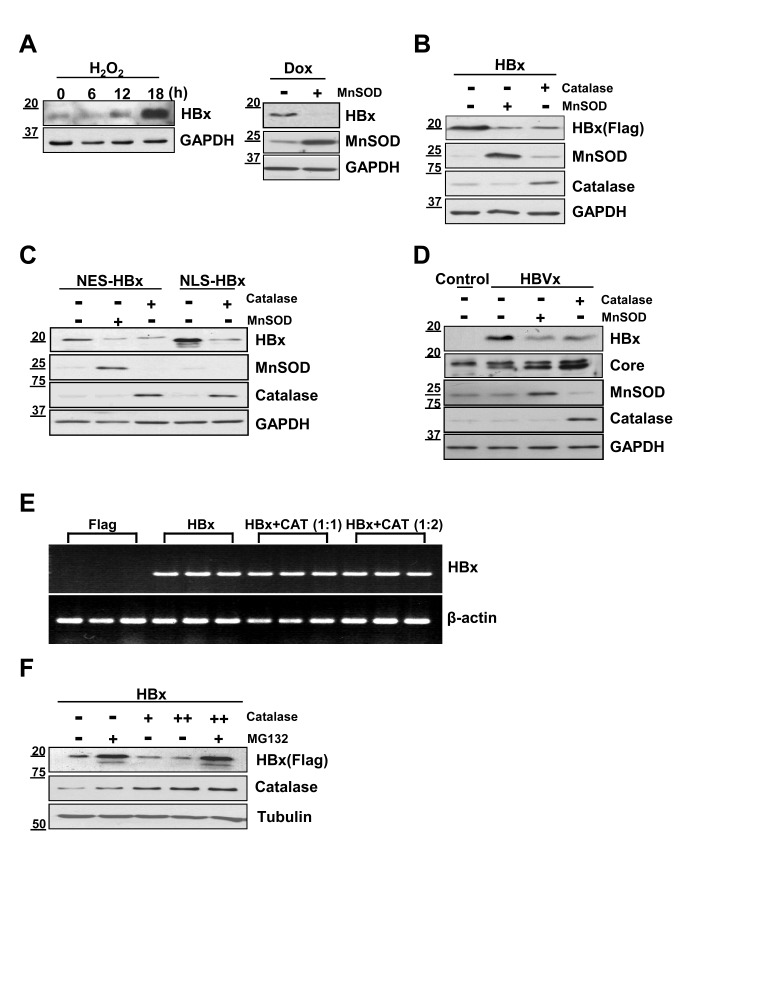
HBx protein levels are decreased by catalase or MnSOD independent of HBx localization (A) Stably HBx-expressing Chang X-34 cells were treated with 0.5 mM H_2_O_2_ in a time-dependent manner. Doxycycline-induced HBx protein in Chang X-34 cells was decreased by the overexpression of MnSOD. (B) Huh7 cells were cotransfected with Myc-tagged HBx plus catalase or MnSOD using the PEI method. (C, D) pNES-HBx, pNLS-HBx or HBV 1.2mer construct were transfected into Huh7 cells along with MnSOD or catalase. (E) Total cellular mRNA from transfected Huh7 cells with HBx and catalase were analyzed for HBx mRNA expression. (F) After Huh7 cells were transfected with HBx and catalase, cells were incubated with or without the proteasome inhibitor, MG132 (20 μM) for 4 h. The HBx expression level was detected by Western blotting.

In our previous work, we found that the HBx level in liver cells was sensitive to ROS levels, such that oxidative stress conditions significantly augmented HBx levels [12, 16]. Here, we examined whether the ROS scavengers, catalase and MnSOD suppressed HBx expression levels. Consistent with our previous findings, hydrogen peroxide (H_2_O_2_) treatment of HBx-expressing ChangX-34 cells, previously established in our laboratory [17], markedly upregulated HBx protein levels 18 h after treatment (Figure 1A, left panel). Notably, introduction of MnSOD into Chang X-34 cells induced a significant reduction in HBx levels (Fig. 1A right panel). We further tested the relationship between ROS and HBx levels by transiently transfecting Huh7 liver cancer cells with the HBx gene and found that HBx levels in these cells were also significantly reduced by MnSOD or catalase (Figure 1B). HBx is prevalent in the cytoplasm, but is also found in the nucleus and mitochondria [18, 19]. We therefore, transfected Huh7 cells with HBx fusion constructs containing a nuclear localization signal (pNLS-HBx) or nuclear export signal (pNES-HBx), which promote translocation into or out of the nucleus of cells, respectively. Regardless of HBx subcellular localization, HBx levels were significantly reduced by overexpression of MnSOD or catalase (Figure 1C). Next, we examined whether antioxidants control the level of HBx protein expressed from the endogenous HBx promoter in the HBV genome. As shown in Figure 1D, HBx expressed from the HBV genome was consistently decreased in MnSOD or catalase overexpressed contexts, in which the core antigen level was not affected.

To examine whether this effect of antioxidants on HBx protein levels occurred at the transcriptional level, we determined HBx mRNA levels by reverse transcription-polymerase chain reaction (RT-PCR) and found that HBx mRNA levels were unchanged (Figure 1E). In contrast, the reduction in HBx accumulation observed in the presence of catalase was completely reversed by treatment with the proteasome inhibitor MG132 (Figure 1F). Taken together, our findings strongly suggest that antioxidants act as powerful regulators that reduce the steady-state level of HBx protein in liver cells at the translational level.

### Cysteine residues of HBx are essential to maintain protein stability and transactivation activity

**Figure 2:**
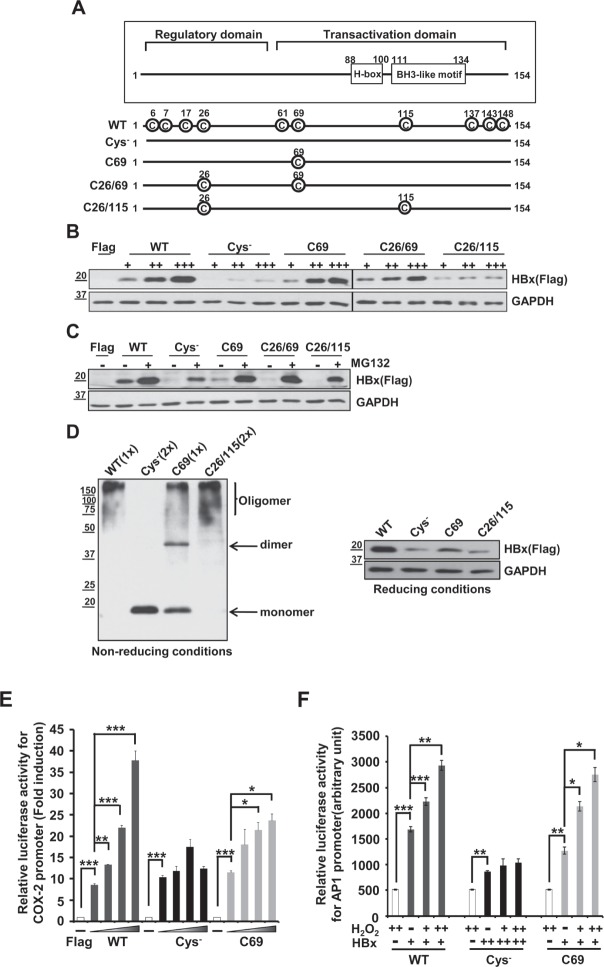
Cys^−^ mutant shows a low stability of HBx protein and attenuates transactivation activity for the COX-2 promoter or AP-1 promoter (A) Schematic diagram showing WT-HBx and four different cysteine mutants of HBx. All the cysteine mutants were generated by replacing Cys to Ser. (B) Huh7 cells were transfected with WT-HBx and four types of cysteine mutants of HBx in a dose-dependent manner. (C) After transfection of WT-HBx and four cysteine mutants into Huh7 cells, 20 μM MG132 was treated on transfected cells for 4 h. (D) WT-HBx and the other three cysteine mutants were overexpressed in Huh7 cells and Western blotting was performed under non-reducing and reducing conditions. (E, F) The COX-2 promoter (−327/+59)-Luc reporter or the AP-1 promoter-Luc reporter construct was transiently transfected with the indicated plasmids (WT-HBx (0.1 μg), Cys^−^-HBx (0.2 μg) or C69-HBx (0.1 μg)) in Huh7 cells. Data shown are mean ± SEM of three independent experiments. Statistically significant differences are indicated: *p<0.05, **p<0.01, ***p<0.005; Student's *t*-test.

HBx has 8 to 10 conserved cysteine residues depending on HBV subtypes [20]. Since HBx level was sensitive to antioxidants, we hypothesized that the stability of HBx protein could be regulated through cysteines, the residues most reactive to intracellular redox status. It is shown that cysteine residues in HBx protein form intra-molecular disulfide bonds as well as inter-molecular disulfide bonds, the latter of which contributes to dimerization of HBx proteins [20, 21]. As a functional motif of HBx, the H-box motif and BH3-like motif have been reported. HBx interacts with the DDB1 of CUL4-DDB1 E3 machinery via its H-box motif and HBx protein stability can be regulated [22]. In addition, HBx binds to Bcl-2 and Bcl-X_L_ through its BH3-like motif, which affects several cellular processes [23]. To test the role of HBx cysteine residues, we first generated four different cysteine mutants: one with no cysteines (Cys^−^), one with a single cysteine remaining at residue 69 (C69), one with two cysteines remaining at residues 26 and 69 (C26/69) and one with two cysteines remaining at residues 26 and 115 (C26/115) (Figure 2A). All the cysteine mutants were generated by replacing Cys to Ser. When these constructs were transfected into Huh7 cells, basal HBx expression levels were quite different among these transfectants (Figure 2B). WT-HBx showed high, stable protein levels, whereas the cysteine mutants showed relatively lower HBx levels. At increasing concentrations (1, 2, 4 μg), both C69 and C26/69 mutant constructs produced stable, dose-dependent accumulation of HBx protein, similar to WT-HBx (Figure 2B). In contrast, the Cys^−^ and C26/115 mutants barely produced HBx protein, suggesting that the cysteine at position 69 is critical for HBx stability. The expression levels of all cysteine mutants were significantly elevated in the presence of MG132 (Figure 2C).

We next examined whether these proteins formed dimers or oligomers. Under non-reducing conditions, the Cys^−^-HBx protein existed exclusively as a monomer, whereas the C69-HBx protein formed not only a monomer but also dimers and oligomers (Figure 2D). On the basis of these results, we postulate that HBx dimer formation requires specific cysteine residues, which likely mediate formation of disulfide linkages between HBx molecules. However, dimeric HBx readily formed oligomers in cells that do not use cysteine residues. Similar to WT-HBx, the C26/115 mutant retaining two cysteine residues primarily formed oligomers.

We previously reported that HBx transactivated the promoter of cyclooxygenase-2 (COX-2), mediator of inflammatory responses [5]. WT-HBx significantly increased the promoter activity of COX-2 in a dose-dependent manner. Both the Cys^−^ and C69 mutants were able to activate the COX-2 promoter; however, dose-dependent increases of luciferase activity were not observed with the Cys^−^ mutant. The C69 mutant also showed a dose-dependent increase in its transactivation function on the COX-2 promoter (Figure 2E). Next, we addressed whether ROS-induced augmentation of HBx levels is correlated to its transactivation function. Since the COX-2 promoter may be activated by H_2_O_2_ through different signaling pathways, we transfected the AP-1 promoter-fused luciferase reporter along with HBx constructs and treated with H_2_O_2_ for 10 h. We found that both wild-type HBx and C69 showed a significant increase in AP-1 luciferase activities. In contrast, there was no increase in luciferase activity with the Cys^−^ mutant (Fig. 2F). Taken together, our data reveal that cysteine residues of the HBx protein are central to maintaining HBx protein stability and its transactivation activity in liver cells.

### Cysteine residues of HBx are important for the HBx-mediated clonogenic cell proliferation

**Figure 3:**
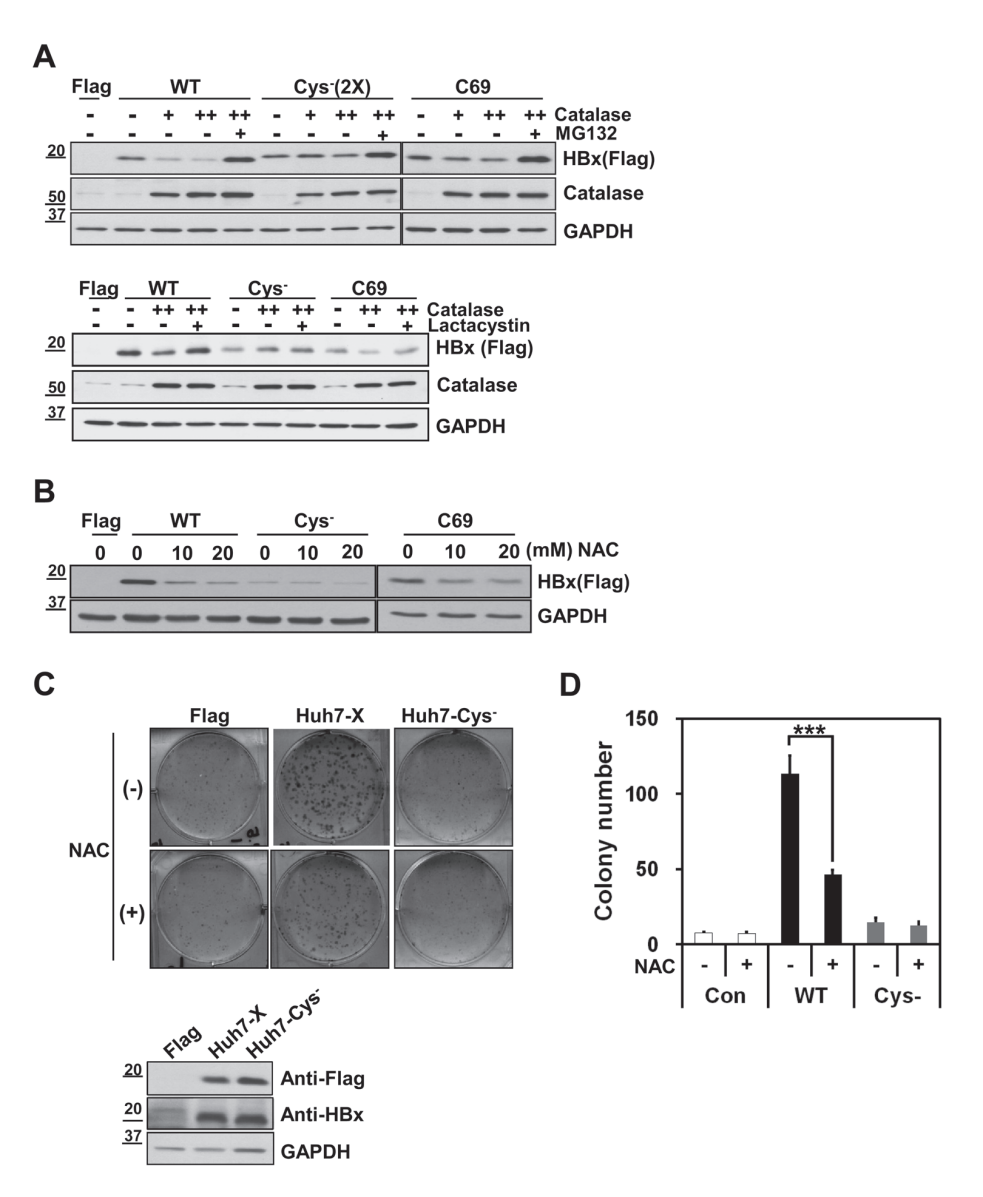
Cysteine residues of HBx are important for HBx protein stability and HBx–mediated cell proliferation (A) Huh7 cells were transiently transfected with either WT-HBx, (0.1 μg), Cys^−^-HBx (0.2 μg) or C69-HBx (0.1 μg) in conjunction with catalase (1 or 2 μg). At lanes 5, 9 and 13, the transfected cells were treated with 20 μM MG132 for 4 h (upper panel). In lower panel, 10 μM Lactacystin was treated into transfected cells for 8 h. (B) After transfection with WT-HBx, Cys^−^-HBx or C69-HBx in Huh7 cells, the transfected cells were treated with N-Acetyl-Cysteine in a dose-dependent manner. (C, D) WT-HBx (0.5 μg) or Cys^−^-HBx (1 μg) plasmids were transfected in Huh7 cells and the transfected cells were selected in the presence of G418. The effect of N-Acetyl-Cysteine on Huh7-X or Huh7-Cys^−^ stable cell lines was determined using clonogenic cell proliferation assay. Data shown are mean ± SEM of three independent experiments. Statistically significant differences are indicated: ***p<0.005; Student's *t*-test.

We next asked whether antioxidants affected the expressions of Cys^−^ and C69 mutants. Since basal expression of the Cys^−^ mutant was weak, we transfected a 2-fold higher concentration of Cys^−^ cDNA than WT-HBx. Notably, expression of the Cys^−^-HBx was barely affected by co-expression of catalase, whereas the basal expression of WT-HBx was consistently reduced (Figure 3A). On the other hand, the levels of the C69-HBx protein were moderately reduced by catalase overexpression. Reduction in HBx levels by catalase was restored by treatment with proteasome inhibitors, MG132 and Lactacystin (Figure 3A), indicating that reduction in HBx levels by catalase occur at the post-translational level. Similar results were obtained by exposing cells to the antioxidant chemical, N-acetyl-cysteine (NAC) (Figure 3B). These findings suggested that HBx cysteine mutants are resistant to antioxidants in maintenance of HBx levels.

Next, we used clonogenic cell proliferation assays to investigate whether the monomeric Cys^−^ mutant exerted an effect on cell function. To this end, we established two stable Huh7 cell lines, one expressing WT-HBx (Huh7-X) and the other expressing Cys^−^-HBx (Huh7-Cys^−^). These cell lines were produced by transfecting Huh7 cells with different concentrations of Flag-tagged WT-HBx or Flag-tagged Cys^−^-HBx and the G418-resistant clones were selected. After determining expression levels of Flag-WT-HBx and Flag-Cys^−^-HBx among different clones by Western blotting (Supplementary Figure S1), we chose two stable clones that showed similar expression levels of HBx (Figure 3C, lower panel). Huh7-X cells generated a significant number of colonies, whereas Huh7-Cys^−^ cells were barely able to form them (Figure 3C, upper panel). Thus, Cys^−^-HBx conferred little in the way of cell proliferation or survival advantage. In addition, NAC treatment reduced the number of Huh7-X cell colonies by more than 50% (Figure 3D), showing that the HBx expression level directly affects cellular behavior. Collectively, in addition to protein stability, cysteine residues of HBx are important for acquiring clonogenic proliferation advantage.

### HBx protein levels are negatively correlated with catalase expression in HBV-related advanced HCC

**Figure 4:**
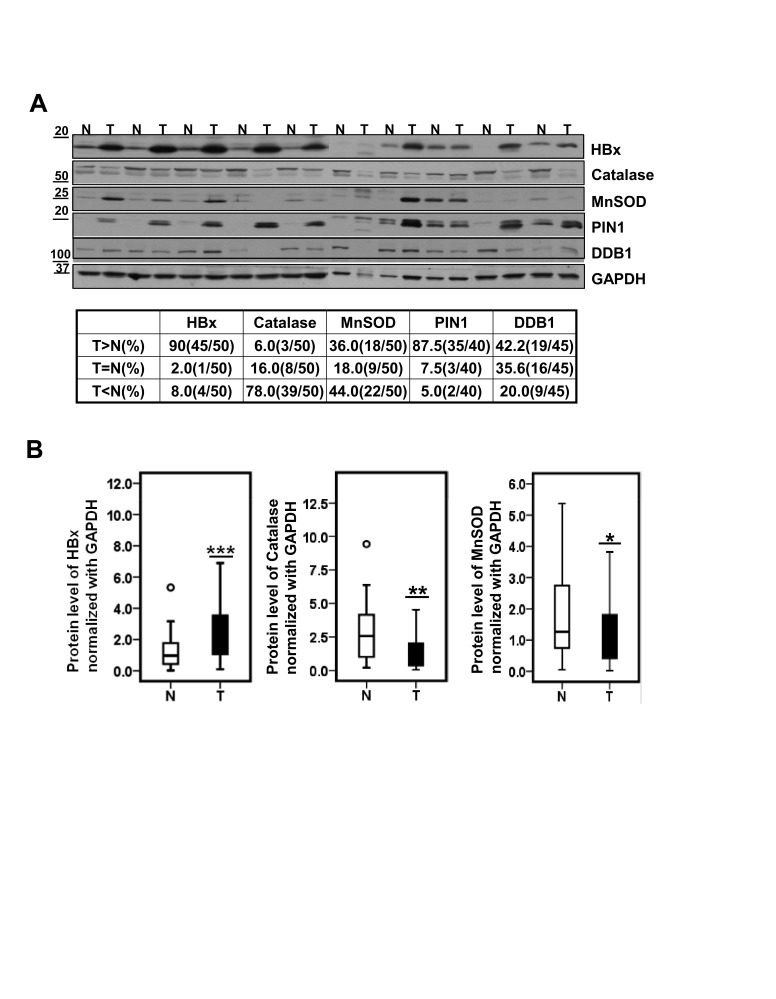
Catalase protein level is significantly lower in HBV-related HCC specimens (A) Representative images of expression level of HBx, catalase, MnSOD, PIN1 and DDB1 in 10 paired HCCs (T) and surrounding non-tumor liver tissues (N) by western blotting. (B) Statistical analysis of HBx, Catalase and MnSOD levels between liver tumors and their surrounding non-tumor tissues. (*p<0.05, **p<0.01, ***p<0.005; Wilcoxon's signed-rank test ).

To address clinical relevance of our findings, we examined whether HBx levels were correlated with antioxidant molecules in HBV-related HCC patients. We first screened more than 100 cases of paired samples of tumor and surrounding non-tumor tissues from HCC patients using Ponceau S staining (Supplementary Figure S2A) or by determining GAPDH (glyceraldehyde-3-phosphate dehydrogenase) expression levels. From these samples we selected 50 paired specimens that showed similar GAPDH expression or protein levels in each sample of the pair. Among 50 paired samples from HCCs patients, 45 (90%) tumor samples showed much higher levels of HBx protein than surrounding non-tumor samples (Figure 4A); conversely, catalase expression level was significantly lower in most tumor samples (78%). In the case of MnSOD, 18 (36%) of 50 paired tissues showed higher expression levels in tumor than in normal whereas 22 (44%) of 50 tissues showed increased expression levels in normal tissues. In addition, PIN1 and DDB1 proteins, also known to regulate HBx protein stability (9-10), were significantly overexpressed in tumor tissues in 35 (87.5%) samples and in 19 (42.2%) samples, respectively (Figure 4A). To further investigate the differences in protein expression level between tumor tissues and non-tumor tissues, we performed densitometric analyses of Western blots and statistical analyses using box plots. Regardless of HCC stages, HBx expression level normalized with GAPDH was about 3 fold higher in tumor tissues than in non-tumor tissues (*** p*<*0.005; Wilcoxon's signed-rank test). Conversely, catalase expression was much lower in tumor tissues than in non-tumor tissues (**p*<*0.01; Wilcoxon's signed-rank test). MnSOD expression also showed a reduction in tumor tissues although this difference was weaker than catalase (Figure 4B).

**Figure 5:**
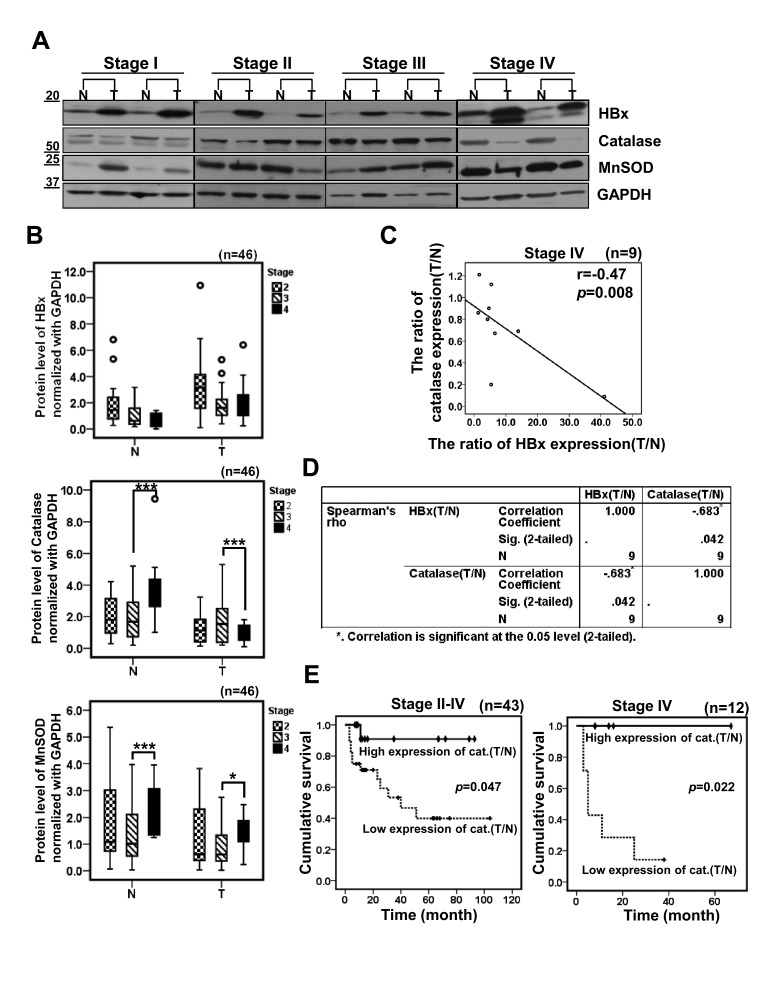
HBx is negatively correlated with catalase in HBV-related advanced HCCs (A) Representative images of Western blotting showing HBx, catalase and MnSOD levels in different cancer stages. (B) Densitometric analysis of HBx, Catalase or MnSOD protein level in surrounding non-tumor and tumor tissues for each cancer stages by Western blotting. (C) A correlation between HBx tumor/non-tumor (T/N) expression ratio and catalase T/N expression ratio in stage IV was examined by scatter plotting in 9 cases of HBV-related HCC patients (r = −0.47, p=0.008; Wilcoxon's signed-rank test). (D) We evaluated a correlation of HBx and catalase using Spearman's rho correlation analysis (p=0.042, coefficient = −0.683). (E) The cumulative overall survival rates of HCC patients by catalase expression level based on Western blotting, analyzed by a Kaplan-Meier curve. p values were determined by the log-rank test.

Next, we analyzed the data by tumor stages and we reached the same conclusion (Figure 5A, 5B), demonstrating that HBx protein levels were all elevated in tumor tissues regardless of cancer stage. In contrast, catalase protein levels were all reduced in tumors. Intriguingly, we have noticed that catalase levels in non-tumor tissues of the stage IV were increased (Figure 5B). In contrast, decrease in catalase level in tumor tissue was especially marked in the stage IV specimens. These findings may suggest that surrounding normal liver tissues at the stage IV cope with the chronic oxidative stresses by elevating catalase levels whereas tumor cells in that stage lose the ability responding to chronic oxidative stresses. Thus, we attempted to analyze these correlations further and calculated the tumor/non-tumor tissue (T/N) ratios of HBx, catalase and MnSOD expression levels for each individual sample by tumor stages (Supplementary Figure S2B). Then, we applied a statistical analysis to establish the correlation between HBx and catalase using their T/N expression ratios. Interestingly, this analysis showed a negative correlation between the T/N expression ratios for HBx and catalase only in stage IV (r = −0.47, p=0.008; Wilcoxon's signed-rank test) (Figure 5C). And a Spearman correlation analysis also showed a strongly negative correlation between HBx and catalase T/N expression ratios in stage IV (Spearman correlation coefficient =-0.683, p=0.042) (Figure 5D). However, we did not observe any correlation between HBx and MnSOD T/N expression ratios in stage IV (Supplementary Figure S3A). Importantly, a Kaplan-Meier analysis showed that the prognosis of HCC patients with lower catalase expression (n=29) was poorer than those with higher catalase expression (n=15) (Figure 5E, left panel). In particular, low catalase expression was significantly associated with shorter overall survival in stage IV HCC patients (p=0.022, Figure 5E, right panel). A Kaplan-Meier analysis of HBx expression showed a trend toward shorter survival in HCC patients with higher HBx expression (n=16) than in those with lower HBx expression (n=28), but this difference did not reach statistically significance (Supplementary Figure S3B). Similarly, MnSOD expression was not associated with patient survival (Supplementary Figure S3C). Together, the T/N ratio of catalase expression in each individual may at least partly reflect its oxidative stress condition. In addition, the T/N ratio of catalase expression in each individual can be used as a better prognostic marker than general catalase expression.

### Prognostic significance of catalase expression in HBV-related advanced HCC

Next we evaluated further a connection between catalase and the clinical features of HCC using in depth statistical analyses. A Univariate Cox regression analysis showed that tumor size, venous invasion, tumor differentiation (Edmondson grade), tumor recurrence, and catalase expression were associated with overall survival (p<0.1; Table 1). A multivariate Cox proportional hazard regression analysis revealed that tumor differentiation (p=0.044) and catalase expression (p=0.035) were independent prognostic factors for the overall survival of HCC patients (Table 2).

**Table 1:** Correlation between survival and clinicopathological characteristics in 44 HCCs

	Survival		
	Dead (n=15)	Alive (n=29)	*P*^a^
Gender			0.815
Male	13	21	
Female	2	8	
AFP (ng/ml)			0.273
≥200	12	16	
<200	3	13	
Tumor size (cm)			0.055
≥5	14	14	
<5	1	15	
Venous Invasion			0.077
Present	12	17	
Absent	3	12	
Modified UICC grade			0.137
Early stage (II)	3	9	
Late stage (III-IV)	12	20	
Edmondson grade			0.065
I/II	4	17	
III/IV	11	12	
Recurrence			0.051
Present	11	10	
Absent	3	17	
Catalase expression ratio (T/N)			0.069
High	1	14	
Low	14	15	
HBx expression ratio (T/N)			0.196
High	7	9	
Low	8	20	
MnSOD expression ratio (T/N)			0.948
High	7	13	
Low	8	16	

a Fisher exact test.

**Table 2:** Effects of catalase expression and clinicopathological characteristics on overall survival of HCC patients in multivariate analysis

Factor	Hazard ratio (95% CI)	*p*^a^
Tumor size (cm)	0.178(0.02-1.596)	0.123
Venous invasion	0.426(0.073-2.505)	0.345
Edmondson grade	4.134(1.041-16.421)	0.044
Recurrence	0.224(0.045-1.124)	0.069
Catalase ratio (T/N)	5.533(1.131-27.079)	0.035

a Fisher exact test.

Finally, to determine whether catalase or HBx expression has clinical significance, we analyzed the correlation between their expression and clinicopathological characteristics. In our cohort, we found no significant correlation between catalase expression and other clinicopathological features (Table 3) as well as HBx expression was not related to clinicopathological features (Table 4). These results imply that the predictive power of catalase is superior to clinicopathological features with respect to overall survival in HCC patients.

**Table 3:** Correlation between catalase expression and clinicopathological characteristics in 44 HCCs

	catalase		
	High expression ratio (T/N) (n=15)	Low expression ratio (T/N) (n=29)	*P*^a^
AFP (ng/ml)			0.749
≥200	8	19	
<200	7	10	
HBx expression ratio (T/N)			0.977
High	5	11	
Low	10	18	
Tumor size (cm)			0.518
≥5	8	20	
<5	7	9	
Tumor multiplicity			0.759
Single	7	17	
Multiple	7	12	
Venous Invasion			0.826
Present	9	12	
Absent	6	17	
Modified UICC grade			0.851
Early stage (II)	4	8	
Late stage (III-IV)	11	21	
Edmondson grade			0.765
I/II	8	13	
III/IV	7	16	
Recurrence			0.927
Present	8	14	
Absent	6	13	
Survival			0.023
Dead	1	14	
Alive	14	15	

a Fisher exact test or Pearson chi-Square.

**Table 4:** Correlation between HBx expression and clinicopathological characteristics in 44 HCCs

	HBx		
	High expression ratio (T/N)	Low expression ratio (T/N)	*P*^a^
	(n=16)	(n=28)	
AFP (ng/ml)			0.789
≥200	11	17	
<200	5	11	
Catalase expression ratio (T/N)			0.977
High	5	10	
Low	11	18	
Tumor size (cm)			0.977
≥5	6	10	
<5	10	18	
Tumor multiplicity			0.954
Single	8	16	
Multiple	7	12	
Venous Invasion			0.341
Present	12	17	
Absent	4	11	
Modified UICC grade			0.488
Early stage (II)	3	9	
Late stage (III-IV)	13	19	
Edmondson grade			0.356
I/II	6	15	
III/IV	10	13	
Recurrence			0.927
Present	8	14	
Absent	6	13	
Survival			0.336
Dead	7	8	
Alive	9	20	

a Fisher exact test or Pearson chi-Square.

## DISCUSSION

For patients with HCC, the prognosis is generally poor and the effectiveness of available remedies is limited. Oxidative stress and associated DNA damage is one of the critical factors involved in the progression of liver cancer [24]. HBV-induced oxidative stress stimulates translocation of the mitogen-activated protein kinase (MAPK) Raf-1 to mitochondria, resulting in phosphorylation of the Raf-1 activation domain. Recently, sorafenib, a multi-kinase inhibitor, was established as the standard of care for patients with advanced HCC for whom loco-regional treatment is not an option [25]. Raf-1 is the key mediator of in the signaling pathway targeted by sorafenib treatment. In addition, our findings here provide a rationale for antioxidant therapeutic approaches in HBV-related HCCs, especially in advanced stages.

We identified catalase as a cellular regulator that effectively reduces the stability of HBx. This is important because HBx cellular levels in liver cells are directly linked to its ability to activate target genes [26] and to stimulate HBV replication and tumorigenic potential [26, 27]. Thus reducing HBx levels could be an important strategy for the prevention and treatment of liver diseases in HBV-infected patients. To date, several regulators of HBx protein stability have been reported. PIN1 has been shown to increase HBx stability [10]. In support of this, we found a strong positive correlation between HBx and PIN1 in HCC tissues (Figure 4A). The current study adds catalase to the list of known HBx modulators, showing that catalase effectively accelerates the degradation of HBx protein (Figure 1). Since chronic HBV infection increases oxidative stress in the liver, it is possible that HBx is accumulated under chronic inflammation conditions due to increased ROS levels. Thus, catalase not only alleviates oxidative stress but also reduces HBx protein levels. Accordingly, we propose that catalase would be more effective than other modulators under chronic oxidative stress conditions.

The effect of catalase on HBx stability involves Cys residues of the HBx protein (Figure 2). The Cys^−^-HBx mutant exclusively formed monomers and showed very little intracellular accumulation. Although the C26/115-HBx mutant mainly formed an oligomer (Figure 2D), its expression levels were very low. In contrast, the C69 mutant protein was readily accumulated, indicating that preservation of the cysteine at residue 69 is important for HBx stability. Notably, C69 lies within the Kunitz domain, which inhibits the function of serine proteases [29]. Since the Kunitz domain is a disulfide-rich structure of α- and β-fold, the disulfide bond at cysteine 69 in HBx may be important for protecting HBx from protease degradation.

We further postulated that catalase expression in HCC patients could be clinically useful for the prediction of patient survival (Figure 5). Here, we used each patient's own non-tumor tissue (N) as a control for catalase expression in tumor (T), calculating a T/N ratio of catalase expression for each patient. We showed for the first time that patients with a relatively high T/N ratio of catalase expression (>0.8) exhibited significantly higher survival than those with low T/N ratios (Figure 5E, Table 3). Thus, the reduction of oxidative stress in tumors by catalase could be crucial for patient survival. This interpretation is supported by others showing that treatment with catalase derivatives significantly reduced the number of metastatic colonies on the surface of the liver [30]. In addition, high levels of ROS and low levels of catalase have been shown to increase tumor progression [31], suggesting that catalase functions as a tumor suppressor. Thus, low catalase expression in tumors compared to non-tumor tissues could serve as a valuable predictor of poor survival of patients with advanced HCC, and enhancement of catalase expression in tumors could be a useful therapeutic strategy for the treatment of HCC. Interestingly, the level of MnSOD, another potent antioxidant molecule that also suppressed HBx accumulation in cells (Figure 1), was not significantly correlated with patient survival (Supplementary Figure S3C).

One of the challenges in the treatment of HCC is the lack of prognostic indicators for patient outcome in advanced cases of HCC. In this study, a low catalase expression level was associated with reduced patient survival, especially in advanced stage IV HCC patients. Furthermore, multivariate analyses showed that catalase expression is an independent factor for patient survival. Considering that the value of prognostic factors in HCC may vary at different disease stages, these results suggest that catalase expression is a potential independent prognostic indicator of overall survival in patients with highly advanced HCC. In the future, additional work is required to clarify the role of oxidative stress in the molecular pathogenesis of advanced HCC and its response to targeted therapy.

## MATERIALS AND METHODS

### Patient's characteristics

Between (June 2005) and (May 2010), over 100 patients with HCC who underwent hepatectomy at Ajou University Hospital were enrolled in this study. A total of 100 paired specimens (tumor and surrounding non-tumor tissues) were obtained after hepatectomy. Of the 100 patients, 44 patients with available follow-up data were eligible for analysis of the association between clinicopathological features and overall survival. All patients were chronic carriers of HBV. Tumor stages were determined according to a modified UICC staging system.

### Statistics

Each experiment was repeated at least three times. Statistical significance was determined by comparing mean values (± standard error of the mean: SEM) using Student's *t*-test and was assumed for p<0.05 (*), p<0.01 (**) and p<0.005 (***). The expression levels of HBx, catalase and MnSOD protein in tumor tissues and surrounding non-tumor tissues were compared using Wilcoxon's signed rank test. Spearman's rho correlation analysis was used to determine the correlation between HBx and catalase protein levels. To further evaluate the prognostic value of the subjects, we determined the cutoff points of the expression ratios (T/N) of catalase and HBx based on western blotting. After measuring the p values of many possible cases, 0.8- and 4.5-fold were taken to be the cutoff values with the minimum p values for catalase and HBx, respectively. In cases with <0.8 T/N expression ratio of catalase in HCC patients, the expression of catalase was considered to be low expression.

Overall survival analysis was estimated using the Kaplan-Meier method. To determine the correlation between catalase or HBx and clinicopathological characteristics, we used either Fisher's exact test or Pearson's chi-square. Data analysis was conducted using the SPSS software (Version 18).

### Cell culture and plasmids

The Huh7 human hepatoma cell line (JCRB0403) and human Chang liver cell line (JCRB9066) were obtained from the Health Science Research Resources Bank (Osaka, Japan) in 1996 and have been maintained in our laboratory. The Chang X-34 cell line was established in our laboratory by transfecting the plasmids of pTetX and pUHD172-1 into the Chang cells [17]. All the cell lines were maintained in Dulbecco's modified Eagle minimal essential medium (DMEM) (GIBCO-BRL, Grand Island, NY), supplemented with 10% fetal bovine serum (FBS). A microbial contamination screening for mycoplasma has been routinely carried out every three months. Hepatic characteristics of these cell lines were authenticated by determining albumin mRNA expression in 2011.

HBx full-length DNA (adr subtype) was amplified from the HBx/pcDNA3.1+ plasmid and subcloned into p3xFLAG-CMV-10 vectors. Four cysteine mutants of HBx were generated by replacing Cys to Ser and subcloned into p3xFLAG-CMV-10 vectors. The HBV 1.2-mer replicon, nuclear localization signal (NLS)-, nuclear export signal (NES)-HBx and human COX-2 promoter linked to luciferase reporter constructs were used as previously described [5].

### RNA extraction, reverse transcription-polymerase chain reaction (RT-PCR)

Total RNA was extracted from hepatoma cells using Trizol reagent (Ambion) according to the manufacturer's instructions. First strand cDNA was synthesized from 1 ug of total RNA by using ReverTra Ace qPCR RT Master Mix (TOYOBO). The expression level of HBx was determined by reverse transcription-PCR using C1000 Touch Thermal Cycler (Bio-rad) and normalized with GAPDH. The primer sets used for HBx were as follows: sense 5′-AGGATCTATGGCTGCTAGGCT-3′ and antisense 5′-GGTACCCTAGGCAGAGGTGAA-3′; for GAPDH, sense 5′-CCATGGAGAAGGCTGGGG-3′ and antisense 5′-CACTGACACGTTGGCAGTGG-3′. Reactions were assayed in triplicate.

### Western blotting analysis

Protein (20 ug) extracted from each of the fresh frozen tumor and surrounding non-tumor liver tissues was resolved in 4-20% SDS-polyacrylamide gels and transferred electrophoretically to nitrocellulose membranes (Bio-rad). Specific antibody for HBx was generated from rabbit. The primary antibodies used were rabbit anti-catalase (Ab Frontier), rabbit anti-MnSOD (Ab Frontier), rabbit anti-GAPDH (Santa Cruz, CA, USA), rabbit anti-Pin1 (Cell Signaling), mouse anti-DDB1 (Santa Cruz, CA, USA), and mouse anti-flag (Sigma).

### Luciferase assays

The transfected cells were harvested at 48 h after transfection. Luciferase assays were performed with the Dual-Luciferase Reporter Assay system (Promega) as instructed by the manufacturer.

### Clonogenic cell proliferation assays

For clonogenicity analysis, 500 or 1000 viable transfected cells (Huh7-X, Huh7-Cys^−^) were seeded in 6-well plates and maintained in complete medium for 2 weeks. Colonies were stained with a mixture of 6% glutaraldehyde and 0.5% crystal violet for 12hrs then rinsed with tap water.

### Funding

This work was supported by the National Research Foundation of Korea(NRF) grant funded by the Korea government(MSIP) (2011-0030043[SRC]).

## SUPPLEMENTARY MATERIAL AND FIGURES



## References

[1] Thun MJ, DeLancey JO, Center MM, Jemal A, Ward EM (2010). The global burden of cancer: priorities for prevention. Carcinogenesis.

[2] Parkin DM, Bray FI, Devesa SS (2001). Cancer burden in the year 2000. The global picture. Eur J Cancer.

[3] Calvisi DF, Ladu S, Gorden A, Farina M, Conner EA, Lee JS, Factor VM, Thorgeirsson SS (2006). Ubiquitous activation of Ras and Jak/Stat pathways in human HCC. Gastroenterology.

[4] Matter MS, Decaens T, Andersen JB, Thorgeirsson SS (2014). Targeting the mTOR pathway in hepatocellular carcinoma: Current state and future trends. J Hepatol.

[5] Lim W, Kwon SH, Cho H, Kim S, Lee S, Ryu WS, Cho H (2010). HBx targeting to mitochondria and ROS generation are necessary but insufficient for HBV-induced cyclooxygenase-2 expression. J Mol Med (Berl).

[6] Lara-Pezzi E, Majano PL, Gomez-Gonzalo M, Garcia-Monzon C, Moreno-Otero R, Levrero M, Lopez-Cabrera M (1998). The hepatitis B virus X protein up-regulates tumor necrosis factor alpha gene expression in hepatocytes. Hepatology.

[7] Na TY, Shin YK, Roh KJ, Kang SA, Hong I, Oh SJ, Seong JK, Park CK, Choi YL, Lee MO (2009). Liver X receptor mediates hepatitis B virus X protein-induced lipogenesis in hepatitis B virus-associated hepatocellular carcinoma. Hepatology.

[8] Kim KH, Shin HJ, Kim K, Choi HM, Rhee SH, Moon HB, Kim HH, Yang US, Yu DY, Cheong J (2007). Hepatitis B virus X protein induces hepatic steatosis via transcriptional activation of SREBP1 and PPARgamma. Gastroenterology.

[9] Bergametti F, Sitterlin D, Transy C (2002). Turnover of hepatitis B virus X protein is regulated by damaged DNA-binding complex. J Virol.

[10] Pang R, Lee TK, Poon RT, Fan ST, Wong KB, Kwong YL, Tse E (2007). Pin1 interacts with a specific serine-proline motif of hepatitis B virus X-protein to enhance hepatocarcinogenesis. Gastroenterology.

[11] Zhao J, Wang C, Wang J, Yang X, Diao N, Li Q, Wang W, Xian L, Fang Z, Yu L (2011). E3 ubiquitin ligase Siah-1 facilitates poly-ubiquitylation and proteasomal degradation of the hepatitis B viral X protein. FEBS Lett.

[12] Wang JH, Yun C, Kim S, Lee JH, Yoon G, Lee MO, Cho H (2003). Reactive oxygen species modulates the intracellular level of HBx viral oncoprotein. Biochem Biophys Res Commun.

[13] Jin YM, Yun C, Park C, Wang HJ, Cho H (2001). Expression of hepatitis B virus X protein is closely correlated with the high periportal inflammatory activity of liver diseases. J Viral Hepat.

[14] Lee YI, Hwang JM, Im JH, Kim NS, Kim DG, Yu DY, Moon HB, Park SK (2004). Human hepatitis B virus-X protein alters mitochondrial function and physiology in human liver cells. J Biol Chem.

[15] Connor KM, Hempel N, Nelson KK, Dabiri G, Gamarra A, Belarmino J, Van De Water L, Mian BM, Melendez JA (2007). Manganese superoxide dismutase enhances the invasive and migratory activity of tumor cells. Cancer Res.

[16] Yun C, Lee JH, Wang JH, Seong JK, Oh SH, Yu DY, Cho H (2002). Expression of hepatitis B virus X (HBx) gene is up-regulated by adriamycin at the post-transcriptional level. Biochem Biophys Res Commun.

[17] Yun C, Lee JH, Park H, Jin YM, Park S, Park K, Cho H (2000). Chemotherapeutic drug, adriamycin, restores the function of p53 protein in hepatitis B virus X (HBx) protein-expressing liver cells. Oncogene.

[18] Sirma H, Weil R, Rosmorduc O, Urban S, Israel A, Kremsdorf D, Brechot C (1998). Cytosol is the prime compartment of hepatitis B virus X protein where it colocalizes with the proteasome. Oncogene.

[19] Rahmani Z, Huh KW, Lasher R, Siddiqui A (2000). Hepatitis B virus X protein colocalizes to mitochondria with a human voltage-dependent anion channel, HVDAC3, and alters its transmembrane potential. J Virol.

[20] Kaveri Sidhu, Saravanan Kumar, Vanga Siva Reddy, Vijay Kumar (2014). Mass spectrometric determination of disulfide bonds in the biologically active recombinant HBx protein of Hepatitis B virus. Biochemistry.

[21] Gupta A, Mal TK, Jayasuryan N, Chauhan VS (1995). Assignment of disulphide bonds in the X protein (HBx) of hepatitis B virus. Biochem Biophys Res Commun.

[22] Ti Li, Eva I Robert, Pieter C van Breugel, Michel Strubin, Ning Zheng (2010). A promiscuous α-helical motif anchors viral hijackers and substrate receptors to the CUL4-DDB1 ubiquitin ligase machinery. Nat Struct Mol Biol.

[23] Hideki Kusunoki, Toshiyuki Tanaka, Toshiyuki Kohno, Kaori Wakamatsu, Isao Hamaguchi (2014). Structural characterization of the BH3-like motif of hepatitis B virus X protein. Biochem Biophys Res Commun.

[24] Nair J, Srivatanakul P, Haas C, Jedpiyawongse A, Khuhaprema T, Seitz HK, Bartsch H (2010). High urinary excretion of lipid peroxidation-derived DNA damage in patients with cancer-prone liver diseases. Mutat Res.

[25] Llovet JM, Ricci S, Mazzaferro V, Hilgard P, Gane E, Blanc JF (2008). Sorafenib in advanced hepatocellular carcinoma. N Engl J Med.

[26] Zhang T, Zhang J, You X, Liu Q, Du Y, Gao Y, Shan C, Kong G, Wang Y, Yang X, Ye L, Zhang X (2012). Hepatitis B virus X protein modulates oncogene Yes-associated protein by CREB to promote growth of hepatoma cells. Hepatology.

[27] Yen CJ, Lin YJ, Yen CS, Tsai HW, Tsai TF, Chang KY, Huang WC, Lin PW, Chiang CW, Chang TT (2012). Hepatitis B virus X protein upregulates mTOR signaling through IKKbeta to increase cell proliferation and VEGF production in hepatocellular carcinoma. PLoS One.

[28] You X, Liu F, Zhang T, Lv N, Liu Q, Shan C, Du Y, Kong G, Wang T, Ye L, Zhang X (2014). Hepatitis B virus X protein upregulates Lin28A/Lin28B through Sp-1/c-Myc to enhance the proliferation of hepatoma cells. Oncogene.

[29] Ranasinghe S, McManus DP (2013). Structure and function of invertebrate Kunitz serine protease inhibitors. Dev Comp Immuno.

[30] Nishikawa M, Tamada A, Hyoudou K, Umeyama Y, Takahashi Y, Kobayashi Y, Kumai H, Ishida E, Staud F, Yabe Y, Takakura Y, Yamashita F, Hashida M (2004). Inhibition of experimental hepatic metastasis by targeted delivery of catalase in mice. Clin Exp Metastasis.

[31] Gupta A, Butts B, Kwei KA, Dvorakova K, Stratton SP, Briehl MM, Bowden GT (2001). Attenuation of catalase activity in the malignant phenotype plays a functional role in an *in vitro* model for tumor progression. Cancer Lett.

