# Supplementary material for: Prognostic significance of catalase expression and its regulatory effects on hepatitis B virus X protein (HBx) in HBV-related advanced hepatocellular carcinomas
Source: Oncotarget. 2014 Oct 24;5(23):12233–46. doi: 10.18632/oncotarget.2625 (PMC4322996; doi:10.18632/oncotarget.2625)
Supplement: Supplementary file 1 [file oncotarget-05-12233-s001.pdf]

# Prognostic significance of catalase expression and its regulatory effects on hepatitis B virus X protein (HBx) in HBV-related advanced hepatocellular carcinomas

**Suppleentary Material**

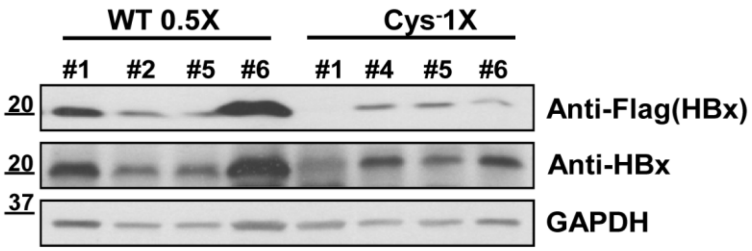


**Figure S1**: **Generation of WT- or Cys$^-$-HBx stable cell lines** To generate stable WT- or Cys$^-$-HBx cell lines, Huh7 cells were transfected with WT-HBx (0.5 μg) or Cys$^-$-HBx (1 μg) and selected in the presence of G418. After 2 weeks, we chose 2 clones, #5 among WT-HBx clones and #6 among Cys$^-$-HBx clones.

A

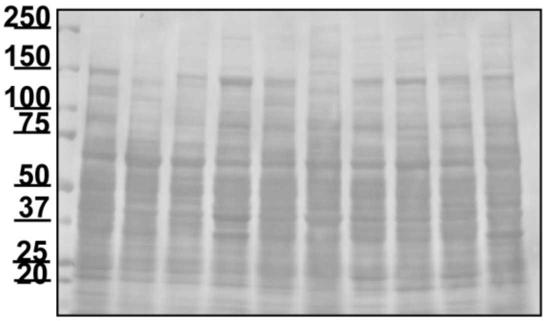


B

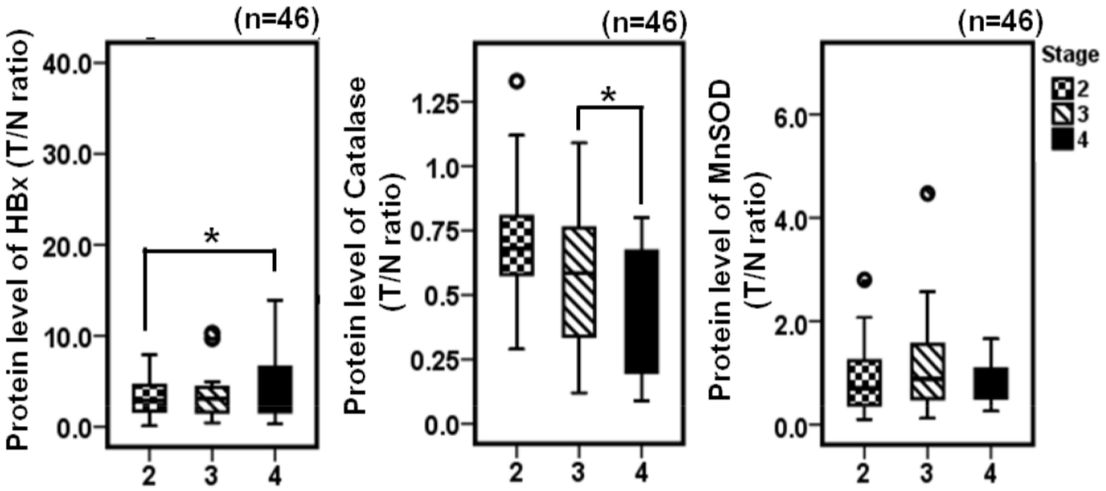


C

| | | | HBx | Catalase |
|---|---|---|---|---|
| Spearman's rho | HBx | Correlation Coefficient | 1.000 | -.079 |
| | | Sig. (2-tailed) | . | .829 |
| | | N | 10 | 10 |
| | Catalase | Correlation Coefficient | -.079 | 1.000 |
| | | Sig. (2-tailed) | .829 | . |
| | | N | 10 | 10 |

**Figure S2: Statistical analysis of HCC patients samples** (A) To compare amounts of protein in tumor and non-tumor tissues in each patient, we screened more than 100 cases of paired samples of tumor and surrounding non-tumor tissues from HCC patients using Ponceau S staining. (B) Densitometric analysis of HBx, Catalase or MnSOD tumor/non-tumor (T/N) expression ratios in each cancer stages. (C) A correlation between HBx and catalase expression level normalized with GAPDH in stage IV using Spearman's rho correlation analysis (p=0.829, coefficient = -0.079).

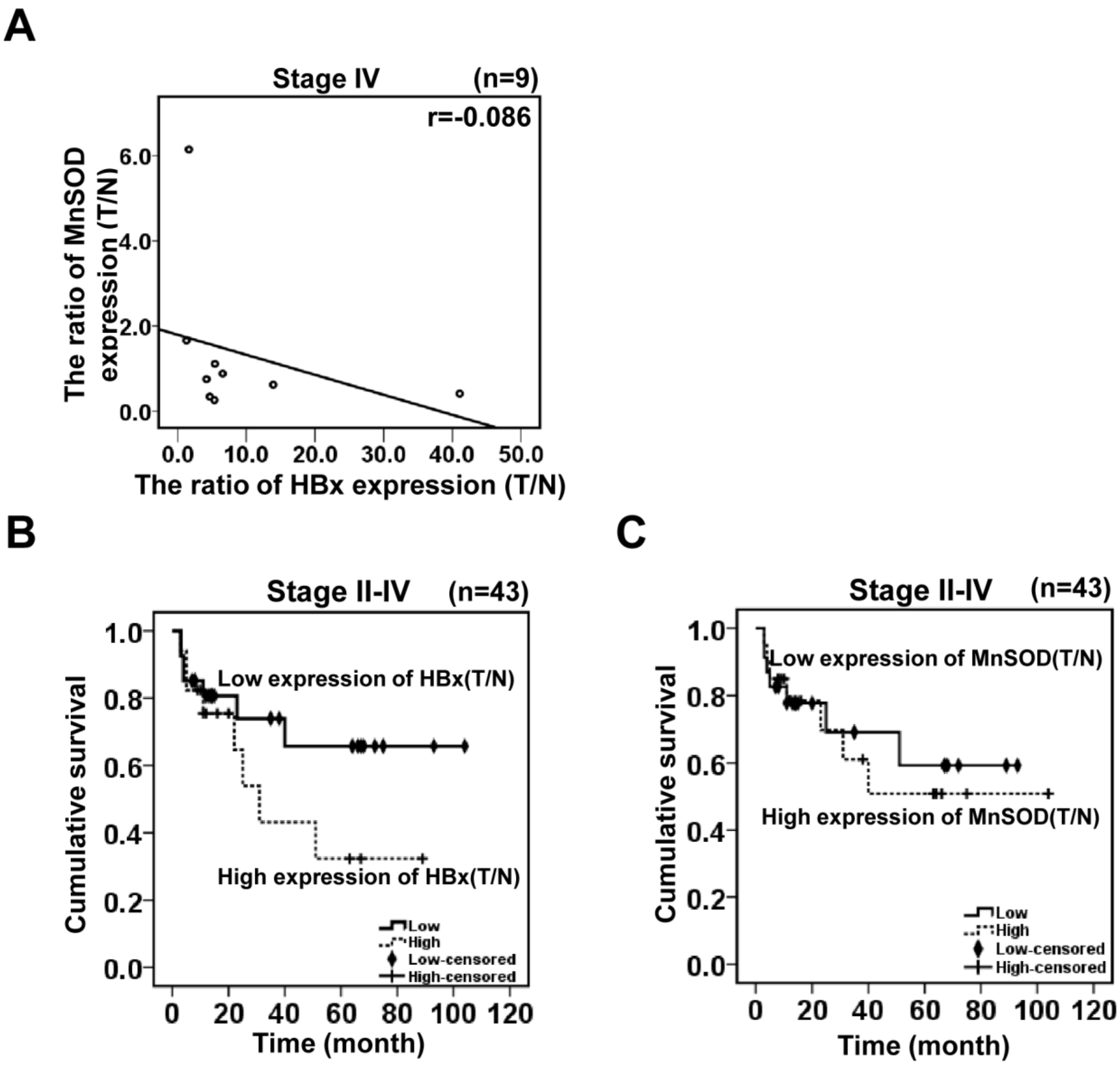


**Figure S3**: **Cumulative overall survival rates of HCC patients by HBx or MnSOD expression level** (A) A correlation between HBx T/N expression ratio and MnSOD T/N expression ratio in stage IV was examined by scatter plotting. Statistically significant differences are indicated: *$p<0.05$ ( Wilcoxon's signed-rank test). (B, C) The cumulative overall survival rates of HCC patients by HBx or MnSOD expression level, analyzed by a Kaplan-Meier curve. p values were not significant
